# Emergence of extensively drug-resistant *Klebsiella pneumoniae* from broiler chickens and clinical samples in Egypt

**DOI:** 10.1186/s12866-025-04187-x

**Published:** 2025-07-31

**Authors:** Mohamed E. Enany, Heba S. El-Mahallawy, Wafaa E. I. Mohammed, Wafaa M. M. Hassan, Abdelkarim M. Aboueisha

**Affiliations:** 1 Department of Bacteriology, Immunology, and Mycology, Faculty of Veterinary Medicine, Suez Canal University, Ismailia, 41522 Egypt; 2 Department of Zoonoses, Faculty of Veterinary Medicine, Suez Canal University, Ismailia, 41522 Egypt; 3 Free Veterinarian, Ismailia, Egypt; 4 Reference Laboratory for Veterinary Quality Control on Poultry Production (Dokki), Animal Health Research Institute, Giza, Egypt

**Keywords:** Antimicrobial resistance, Chicken, Human, *K. pneumoniae*, Virulence genes

## Abstract

**Background:**

*Klebsiella pneumoniae* (*K. pneumoniae*) is an opportunistic pathogen belonging to *Enterobacteriaceae* family. It is present in the intestinal tract of animals and humans, and is considered as a major threat to public health. A total of 33 previously identified *K. pneumoniae* strains isolated from of broiler chicken (*n* = 20) and, humans (*n* = 13) were tested for their antimicrobial sensitivity and virulence genes detection.

**Results:**

The antimicrobial sensitivity testing of broiler chickens *K. pneumoniae* strains, showed complete phenotypic resistance towards ampicillin (100%), colistin (100%), fosfomycin (100%), trimethoprim/sulphamethaxole (100%) followed by gentamicin (90%), cefotaxime (90%) and chloramphenicol (75%). However, all the isolates were sensitive to imipenem (100%). *K. pneumoniae* human strains showed complete phenotypic resistance to fosfomycin (100%), followed by ampicillin (92.3%), colistin (92.3%), and ciprofloxacin and trimethoprim/sulfamethaxole (76.9%, each). The resistance gene profiling of the chicken strains was *cml*A (65%), *dfr*A1 (65%), *mcr*1 (60%), *tet*A (50%), *fos*A (40%), and *sul*1 (35%), and the most prevalent virulence gene was *rmp*A (85%), that was detected either alone in 9 isolates (52.9%), or in combination with the *mag*A gene in 8 isolates (47.1%), and with *fim*H gene in 8 isolates (47.1%), followed by *fim*H (55%) and *mag*A genes (40%). On the other hand, the human strains gene profiling showed *mcr*1 (61.5%), *cml*A (53.8%), and *sul*1 (46.2%) genes, while *dfr*A1 was not detected. The most prevalent virulence genes in the human strains were *rmp*A and *mag*A genes (46.2%, each), followed by *fim*H gene (38.5%). Overall, multidrug-resistant (MDR) (33.3%), extensively-drug resistant (XDR) (57.6%) and pan-drug resistant (PDR) (9.1%) isolates were detected.

**Conclusion:**

The distribution of such virulent and resistant *K. pneumoniae* isolates among apparently healthy broiler chicken, highlights their potential role in the transmission of both resistance and virulence attributes to the surrounding environment and human contacts. Moreover, spreading of carbabenem resistant *K. pneumoniae* among human isolates, suggest the urgent need for more strict measures for the uncontrolled usage of antibiotics.

## Introduction

Food producing animals; including poultry; are thought to be a major reservoir of multidrug-resistant (MDR) microorganisms [1]. The use of antimicrobials in broiler chickens is usually higher than that in laying hens, due to the need for maintaining a rapid growth of broilers [2]. The resistance to antimicrobials is not only confidential to pathogenic bacteria but also occurs in the normal flora of animals and humans, consequently, chicken endogenous flora may travel across the food chain and temporarily inhabit the human digestive system [3, 4]. *K. pneumoniae* is one of the most significant global sources of multi-drug resistant (MDR) infections, leading to increased healthcare costs and high mortalities [5]. Recently, the rate of resistance to colistin in *Enterobacteriaceae*, especially *K. pneumoniae*, has augmented. Due to the extensive use of antibiotics in humans, veterinary medicine, and agricultural practices during the last few decades, the emergence of *K. pneumoniae* strains that harbor various resistance genes has increased [6].

In humans, *K. pneumoniae* is a one health pathogen that has been identified as one of the most popular causes of infections developed in hospitals and the community, being responsible for bloodstream infections, wound infections, pneumonia, and meningitis [7, 8]. About 2 to 5% of community-acquired pneumonia have been linked to *K. pneumoniae*, which is the most common cause of lung infections in healthy individuals [9]. Throughout time, antibiotic-resistant *K. pneumoniae* strains have been produced in hospitals due to the widespread use of antibiotics, leading to limited available treatment options for medical intervention against *Klebsiella* infections [10]. The hospitalized patients have a much higher carrier prevalence for *K. pneumoniae* than those in the community. For instance, one study has documented a link between the amount of antibiotics given and the carrier rates, which can reach 77% in the feces of hospitalized patients [11].

The spreading of *K. pneumoniae* has become a threat to public health especially after the emergence of multi-drug resistant (MDR) isolates [12]. The spread of *K. pneumoniae;* which has carbapenem and third-generation cephalosporin resistance; is of particular concern since it causes illnesses with few effective treatment options [13].

The pathogenicity of *K. pneumoniae* derived from hospital acquired infections is significantly influenced by the virulence associated genes, which include the coding regulators of mucoid phenotype A (*rmp*A) [14]. *K. pneumoniae* pathogenesis is associated with the presence of certain virulence genes that encode virulence factors, which allow the bacteria to attack the mammalian immune system and lead to a variety of diseases [15]. *K. pneumoniae* is currently recognized as one of the ESKAPE organisms, and despite its reporting in various human researches worldwide [16, 17], and in Egypt [18, 19], there was no enough veterinary data about this topic. Moreover, without continuous monitoring of the antibiotic resistance in *K. pneumoniae*, the treatment would be incredibly difficult. Therefore, the aim of the present study was to obtain a better understanding of the antimicrobial sensitivity and the molecular characteristics of *K. pneumoniae* from apparently healthy broiler chicken and human patients, and to highlight its public health significance in Ismailia city, Egypt.

## Materials and methods

### Ethical statement

Sample processing and experiments for the present study have been reviewed and approved by the Ethical Committee of the Scientific Research Board, Faculty of Veterinary Medicine, Suez Canal University, Ismailia, Egypt (No. 2019032).

### Samples

**Fig. 1:**
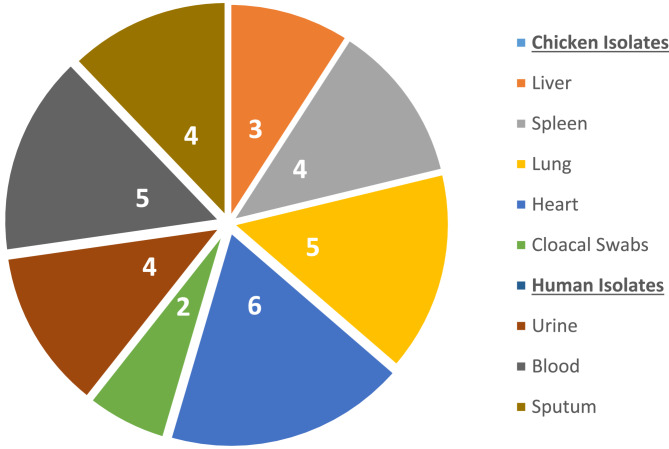
Number and sources of *K. pneumoniae* isolates tested in this study

A total of 33 previously identified *K. pneumoniae* isolates of clinically healthy broiler chicken (*n* = 20) [20] and human patients (*n* = 13), were tested for their antimicrobial sensitivity using disc diffusion method, and by molecular techniques (PCR) for detection of antimicrobial resistance and virulence genes. The chicken isolates were collected from liver, spleen, heart, lung, and cloacal samples of apparently healthy chickens in live bird market, that are ready to be sold for consumers, while human isolates originated from blood, urine, and sputum samples of inpatients hospitalized with various infections of chest, blood and urinary tract, in a governmental hospital in Ismailia City (Fig. 1). Oral informed consent was obtained from each patient to use the obtained isolates in the study under the supervision of the laboratory manager. The patients’ privacy were protected by keeping participants anonymous, and their private data were not acquired or used in the research.

### Antimicrobial sensitivity testing of *K. pneumoniae* isolates

*K. pneumoniae* isolates were tested for their antimicrobial sensitivity against 13 antimicrobials from different groups using the disc diffusion technique on Muller Hilton agar (MHA) (HiMedia India). Antimicrobial discs (Oxoid, UK) with the following concentrations were used: ampicillin (AM) 10 µg, imipenem (IPM) 10 µg, amoxicillin/clavulanic acid (AMC) 30 µg, ceftriaxone (CTR) 30 µg, cefotaxime (CTX) 30 µg, chloramphenicol (C) 30 µg, ciprofloxacin (CIP) 5 µg, colistin (CT) 10 µg, fosfomycin (FF) 50 µg, gentamicin (Gen) 10 µg, trimethoprim-sulfamethoxazole (SXT) 25 µg, and tetracycline (TE) 30 µg. Few colonies of similar morphology of each *K. pneumoniae* isolate were transferred using a sterile loop to a test tube containing 5 ml sterile Muller Hilton broth (HiMedia India). Addition of sterile saline or other colonies to the tube was applied until the turbidity was adjusted to match 0.5 McFarland standard tubes against adequate light. Standardized suspensions were evenly streaked on Muller Hilton agar (MHA) plates using sterile cotton swabs and left for 3–5 min. before antimicrobial discs were applied firmly using sterile forceps. The plates were then incubated at 37^o^C for 18 h and the inhibition zones around different discs were measured in mm [21].

### Detection of the resistance pattern of *K. pneumoniae* isolates

 The isolates were designated as multidrug-resistant (MDR) if they were resistant to at least one agent in 3 or more antimicrobial classes. If isolates showed resistance to most of the tested antimicrobial classes except one or two classes, they were categorized as extensively-drug resistant (XDR). However, the isolates were classified as pan-drug resistant (PDR) when they showed complete resistance to all agents in the tested antimicrobial classes [22].

### Molecular characterization of *K. pneumoniae* isolates

 Plasmid DNA Miniprep extraction kits (Thermo Fisher Scientific, Waltham, MA, USA) and QIAamp DNA mini kit (Qiagen, Germany, GmbH) were used to extract DNA for detection of the resistance and virulence genes, respectively, according to the manufacturer instructions. Six µl extracted DNA of each isolate was used in each 25 µl PCR reaction volume using 12.5 µl of 2× Emerald Amp Master Mix (Takara, Japan), and 1 µl of each specific forward and reverse primer (Metabion, Germany) for each target gene as mentioned in Table 1. PCR amplification was performed in a programmable thermal controller (Perkin Elmer/Cetus Research, USA). PCR products (20 µl) were electrophoresed in 1.5% agarose (ABgene) gel using 1× TBE buffer before being photographed by a gel documentation system. The positive controls used were reference strains kindly supplied form the Animal Health Research Institute, Zagazig city, Egypt.

**Table 1:** Oligonucleotide primer sequences of antimicrobial resistance and virulence genes for *K. pneumoniae*

Target gene	Primers sequences	Amplified product(bp)	Primary den.	Sec. den.	Ann.	Ext.	Final ext.	Reference
*cml*A	TACTCGGATCCATGCTGGCC	578	94˚C 5 min.	94˚C 30 s.	65˚C 40 s.	72˚C 45 s.	72˚C 10 min	[23]
	TCCTCGAAGAGCGCCATTGG							
*fos*A	ATCTGTGGGTCTGCCTGTCGT	271	94˚C 5 min.	94˚C 30 s.	50˚C 40 s.	72˚C 45 s.	72˚C 10 min	[24]
	ATGCCCGCATAGGGCTTCT							
*dfr*A1	TGGTAGCTATATCGAAGAATGGAGT	425	95˚C 10 min.	95˚C 15 s.	60˚C 1 min.	72˚C 30 s.	72˚C 10 min.	[25]
	TATGTTAGAGGCGAAGTCTTGGGTA							
*tet*A	GGCGGTCTTCTTCATCATGC	502	95˚C 10 min.	95˚C 15 s.	60˚C 1 min.	72˚C 30 s.	72˚C 10 min.	[26]
	CGGCAGGCAGAGCAAGTAGA							
*sul*1	CC GATATTGCTGAGGCGG	435	95˚C 10 min.	95˚C 15 s.	60˚C 1 min.	72˚C 30 s.	72˚C 10 min.	[27]
	CCAACGCCGACTTCAGCT							
*mcr*1	CGGTCAGTCCGTTTGTTC	305	95˚C 10 min.	95˚C 15 s.	60˚C 1 min.	72˚C 30 s.	72˚C 10 min.	[28]
	CTTGGTCGGTCTGTAGGG							
*mag*A	GGTGCTCTTTACATCATTGC	1282	94˚C 5 min.	94˚C 2 min.	60˚C 1 min.	72˚C 1 min.	72˚C 3 min.	[29]
	GCAATGGCCATTTGCGTTAG							
*rmp*A	ACTGGGCTACCTCTGCTTC	516	94˚C 5 min.	94˚C 2 min.	60˚C 1 min.	72˚C 1 min.	72˚C 3 min.	
	CTTGCATGAGCCATCTTTCA							
*fim*H	TGCTGCTGGGCTGGTCGATG	688	94˚C 5 min.	94˚C 2 min.	60˚C 1 min.	72˚C 1 min.	72˚C 3 min.	[30]
	GGGAGGGTGACGGTGACATC							

### Statistical analysis

 Analysis and data visualization were performed using Microsoft Excel 2016, and corrplot and GGally packages of R-software version 4.3.1. were used to study the correlation between different genes. Data were considered significant at *P* values ≤ 0.05.

## Results

**Fig. 2:**
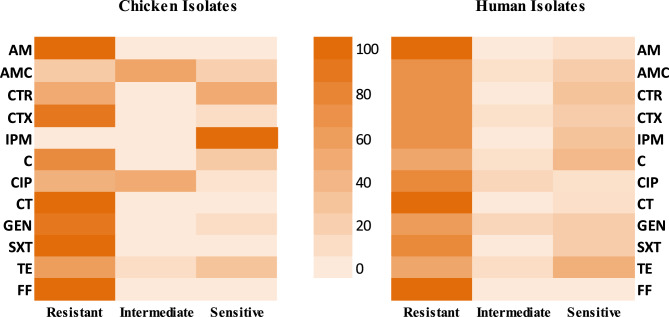
Heatmap of the resistance of *K. pneumoniae* isolates from broiler chicken and human to different antimicrobial agents

In this study, the antimicrobial susceptibility testing of *K. pneumoniae* isolates from broiler chicken, showed complete resistance to ampicillin, colistin, fosfomycin, and trimethoprim/sulfamethoxazole (100%, each), with a very high resistance to gentamicin, cefotaxime (90%, each), and chloramphenicol (75%), and moderate resistance levels against tetracycline (60%), ceftriaxone (50%), and ciprofloxacin (45%). Notably, a low resistance level was reported against amoxicillin-clavulanic acid (25%). However, all the isolates were still sensitive to imipenem (100%) (Table 2; Fig. 2).

**Table 2:** Antimicrobial susceptibility test results of *K. pneumoniae* isolates from apparently healthy broiler chicken

Antimicrobial class	Antimicrobial agent and concentration	Resistant No. (%)		Intermediate No. (%)	Sensitive No. (%)
Penicillins	Ampicillin (10 µg)	20 (100)	0 (0.00)		0 (0.00)
Β-lactams combination agents	Amoxicillin/clavulanic acid (30 µg)	5 (25)	11 (55)		4 (20)
Cephems	Ceftriaxone (30 µg)	10 (50)	0 (0.00)		50 (50)
	Cefotaxime (30 µg)	18 (90)	0 (0.00)		2 (10)
Penems	Imipenem (10 µg)	0 (0.00)	0 (0.00)		20 (100)
Phenicols	Chloramphenicol (30 µg)	15 (75)	0 (0.00)		5 (25)
Fluoroquinolones	Ciprofloxacin (5 µg)	9 (45)	10 (50)		1 (5)
Lipopeptides	Colistin (10 µg)	20 (100)	0 (0.00)		0 (0.00)
Aminogylcosides	Gentamicin (10 µg)	18 (90)	0 (0.00)		2 (10)
Folate pathway antagonists	Trimethoprim- Sulfamethoxazole (25 µg)	20 (100)	0 (0.00)		0 (0.00)
Tetracyclines	Tetracycline (30 µg)	12 (60)	2 (10)		6 (30)
Fosfomycins	Fosfomycin (50 µg)	20 (100)	0 (0.00)		0 (0.00)

Human strains showed complete antibiotic resistance to fosfomycin (100%), with very high resistance to ampicillin (92.3%), colistin (92.3%), trimethoprim/sulfamethoxazole (76.9%), ciprofloxacin (76.9%), cefotaxime, imipenem, ceftriaxone, and amoxacillin-clavulinic acid (69.2%, each). A moderate antibiotic resistance levels were recorded against gentamicin (61.5%), chloramphenicol, and tetracycline 53.8%, each (Table 3,Fig. 2.

**Table 3:** Antimicrobial susceptibility test results of *K. pneumoniae* isolates from human

Antimicrobial class	Antimicrobial agent and concentration	Resistant No. (%)	Intermediate No. (%)	Sensitive No. (%)
Penicillins	Ampicillin (10 µg)	12 (92.3)	0 (0.00)	1 (7.7)
Β-lactams combination agents	Amoxcillin/clavulanic acid (30 µg)	9 (69.2)	1 (7.7)	3 (23.1)
Cephems	Ceftriaxone (30 µg)	9 (69.2)	0 (0.00)	4 (30.8)
	Cefotaxime (30 µg)	9 (69.2)	1 (7.7)	3 (23.1)
Penems	Imipenem (10 µg)	9 (69.2)	0 (0.00)	4 (30.8)
Phenicols	Chloramphenicol (30 µg)	7 (53.8)	1 (7.7)	5 (38.5)
Fluoroquinolones	Ciprofloxacin (5 µg)	10 (76.9)	2 (15.4)	1 (7.7)
Lipopeptides	Colistin (10 µg)	12 (92.3)	0 (0.00)	1 (7.7)
Aminogylcosides	Gentamicin (10 µg)	8 (61.5)	2 (15.4)	3 (23.1)
Folate pathway antagonists	Trimethoprim- Sulfamethoxazole (25 µg)	10 (76.9)	0 (0.00)	3 (23.1)
Tetracyclines	Tetracycline (30 µg)	7 (53.8)	0 (0.00)	6 (46.2)
Fosfomycins	Fosfomycin (50 µg)	13 (100)	0 (0.00)	0 (0.00)

**Fig. 3:**
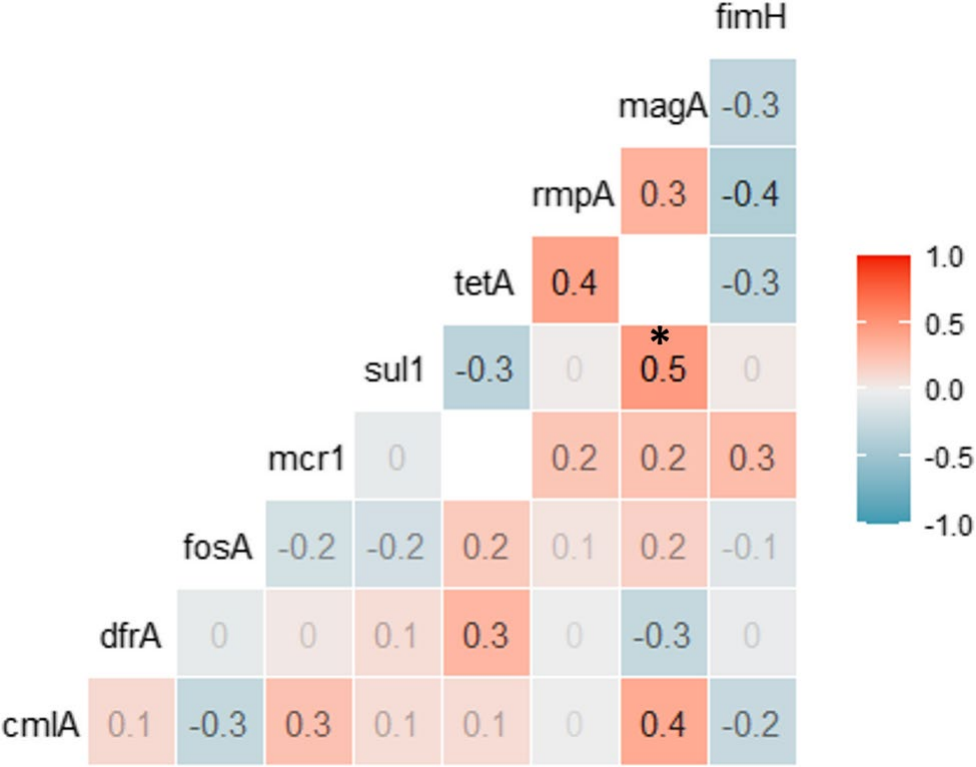
Correlation between detection of virulence and antimicrobial resistance genes in *K. pneumoniae* isolates from broiler chickens. *denotes significant positive correlation (*P< 0.05*)

Resistance and virulence gene profiling of the 33 *K. pneumoniae* isolates is shown in Table (4). In chicken, the most frequent resistance genes were *cml*A (13/20, 65%), *dfr*A1 (13/20, 65%), *mcr*1 (12/20, 60%), and *tet*A (10/20, 50%). The *rmp*A gene was detected in 17 isolates (85%), either alone (in 5 isolates) or in combination with the *mag*A gene (in 8 isolates) and with the *fim*H gene (in 8 isolates). The *fim*H gene was detected in 11 isolates (55%) and the *mag*A gene was detected in 8 isolates (40%). There was a significant direct moderate correlation between *sul*1 and *mag*A genes (*r* = 0.5, *P*<0.05*) (Fig. 3).

**Table 4:** Antimicrobial resistance and virulence genes of *K. pneumoniae* isolates from broiler chicken and human

Source of isolates	No. of isolates	Antimicrobial resistance genes						Virulence genes		
		cmlA No. (%)	dfrA1 No. (%)	fosA No. (%)	mcr1 No. (%)	sul1 No. (%)	tetA No. (%)	rmpA No. (%)	magA No. (%)	fimH No. (%)
Broiler chicken	20	13 (65)	13 (65)	8 (40)	12 (60)	7 (35)	10 (50)	17 (85)	8 (40)	11 (55)
Human	13	7 (53.8)	0 (0.0)	3 (23.1)	8 (61.5)	6 (46.2)	2 (15.4)	6 (46.2)	6 (46.2)	5 (38.5)

*cmiA* chloramphenicol resistance gene, *dfrA* trimethoprim resistance gene, *fos*A fosfomycin resistance gene, *sul*1 sulfonamide resistance gene, *mcr*1 mobile colistin resistance gene, *tetA* tetracycline resistance gene, *fim*H adhesive subunit of type 1fimbriae, *mag*A mucoviscosity-associated gene A, *rmpA* regulator of the mucoid phenotype A gene

**Fig. 4:**
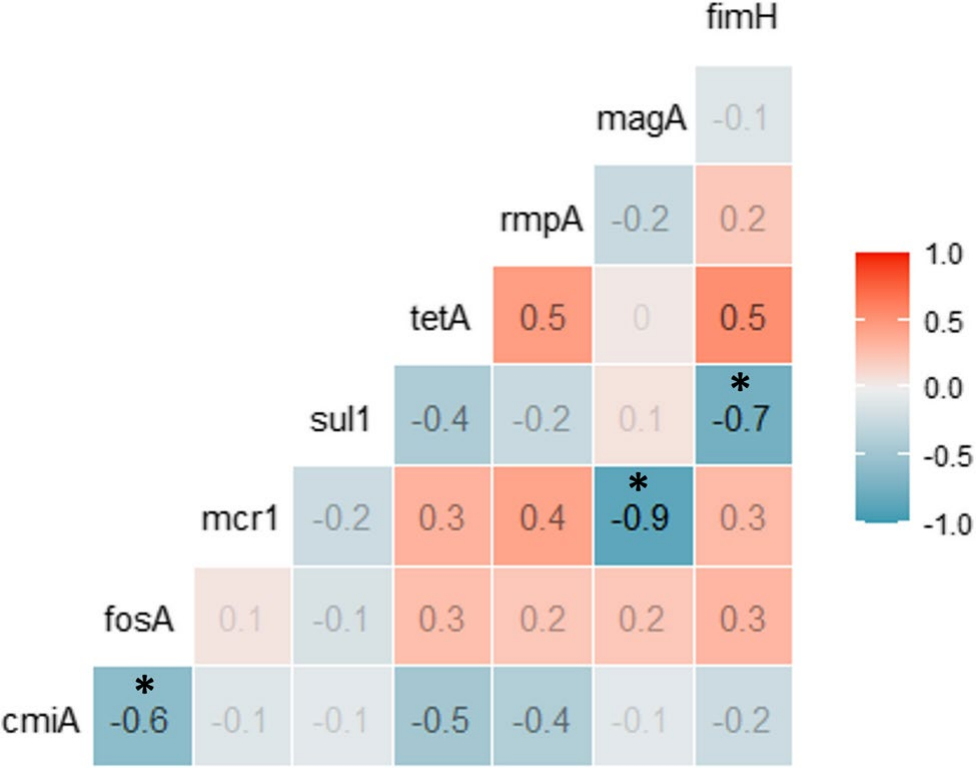
Correlation between detection of virulence and antimicrobial resistance genes in *K. pneumoniae* isolates from human. *denotes significant negative correlation (*P< 0.05 P*)

However, *dfr*A1 could not be detected in any of the human strains, although (76.9%) of them showed a phenotypic resistance to trimethoprim-sulfamethoxazole on the plate. The most frequent resistance genes were *mcr*1 (8/13, 61.5%), *cml*A (7/13, 53.8%), and *sul*1 (6/13, 46.2%). Out of the 13 tested human strains, the *fim*H gene was detected in 5 isolates (38.5%), while both *rmp*A and *mag*A genes were detected each in 6 isolates (46.2%, each). There was a significant strong negative correlation between *mcr*1 and *mag*A genes, and *sul*1 and *fim*H genes (*r*= -0.7, -0.9, *P*<0.01*). However, there was a significant negative moderate correlation between *fos*A and *cmi*A genes (*r*= -0.6, *P*< 0.05*) (Fig. 4).

Overall, out of the 33 tested isolates, (33.3%) of the isolates were MDR, (57.6%) were XDR and (9.1%) were PDR (Table 5).

**Table 5:** Complete resistance and virulence gene profiles of *K. pneumoniae* isolates from apparently healthy broiler chicken and human

	Isolate No.	Source of the isolate	Antimicrobial resistance profile						Virulence profile			Resistance pattern
			*cml*A	*dfr*A	*fos*A	*mcr*1	*sul*1	*tet*A	*rmp*A	*mag*A	*fim*H	
Chicken isolates	1	Heart	+	-	+	-	-	+	+	+	-	XDR
	2	Spleen	+	-	+	+	-	-	+	+	-	MDR
	3	Liver	+	-	-	+	+	-	+	+	-	XDR
	4	Liver	+	-	-	+	+	-	+	+	+	XDR
	5	Lung	+	+	-	+	-	+	+	+	-	XDR
	6	Lung	+	+	-	+	-	-	+	-	+	XDR
	7	Lung	+	+	-	+	-	-	-	-	+	MDR
	8	Lung	+	+	-	+	-	+	+	-	-	XDR
	9	Spleen	-	+	+	-	-	+	+	-	-	XDR
	10	Heart	-	+	-	-	+	-	+	-	-	MDR
	11	Heart	+	+	-	-	+	-	-	-	+	MDR
	12	Spleen	+	+	+	-	-	+	+	-	-	XDR
	13	Heart	-	-	-	+	-	+	+	-	+	XDR
	14	Liver	-	-	-	-	-	-	+	-	+	MDR
	15	Heart	-	-	+	-	-	-	-	-	+	XDR
	16	Heart	-	+	+	+	-	+	+	-	+	MDR
	17	Lung	+	+	-	+	-	+	+	-	+	XDR
	18	Cloacal swab	+	+	+	+	+	+	+	+	+	XDR
	19	Spleen	+	+	-	-	+	+	+	+	-	XDR
	20	Cloacal swab	-	+	+	+	+	-	+	+	+	XDR
Human isolates	21	Urine	-	-	-	+	-	+	+	-	+	MDR
	22	Sputum	-	-	+	+	-	+	+	+	+	MDR
	23	Urine	-	-	-	-	+	-	+	+	-	MDR
	24	Sputum	-	-	+	-	-	-	-	+	+	XDR
	25	Sputum	+	-	-	-	+	-	-	+	-	MDR
	26	Blood	+	-	-	-	-	-	-	+	-	XDR
	27	Blood	+	-	-	-	+	-	-	+	-	PDR
	28	Blood	+	-	-	+	+	-	-	-	-	PDR
	29	Blood	-	-	-	+	+	-	-	-	-	MDR
	30	Blood	-	-	+	+	+	-	+	-	-	PDR
	31	Urine	+	-	-	+	-	-	+	-	-	XDR
	32	Sputum	+	-	-	+	-	-	-	-	+	XDR
	33	Urine	+	-	-	+	-	-	+	-	+	XDR

AM (Ampicillin), AMC (Amoxcillin/clavulanic acid), CTR (Ceftriaxone), CTX (Cefotaxime) IPM (Imipenem), C (Chloramphenicol), CIP (Ciprofloxacin), CT (Colistin), GEN (Gentamicin) SXT (Trimethoprim- Sulfamethoxazole), TE (Tetracycline), FF (Fosfomycin).

## Discussion

This study reported the virulence and antimicrobial resistance of *K. pneumoniae* from apparently healthy broiler chicken and human clinical samples in Ismailia, Egypt. Presence of such resistant and virulent *K. pneumoniae* isolates from broiler chickens’ lungs, hearts, spleens, and liver indicate concurrent extraintestinal infection that threatens public health [31]. In the present study, although chicken isolates were MDR and XDR, they were still completely sensitive to imipenem. Nearly similar findings were reported, where *K. pneumoniae* isolates were sensitive to imipenem (82%) [32]. Also, most of the strains isolated from animals including chicken samples were sensitive to carbapenems as imipenem [33].

On the other hand, *K. pneumoniae* chicken isolates showed complete resistance to ampicillin. Similar findings were reported earlier [32, 34–37]. This may be due to the fact that all *K. pneumoniae* strains, including hypervirulent *K. pneumoniae* (hvKp), are intrinsically resistant to ampicillin [36]. Although tetracycline is a frequently used antibiotic in Egypt and globally due to its efficacy as a broad-spectrum antibiotic that is quickly absorbed, inexpensive, and has few adverse effects [35], a moderate resistance (60%) to this antimicrobial was observed in the present study.

In this work, all *K. pneumoniae* chicken isolates were phenotypically resistant to colistin. The emergence of colistin resistance in *K. pneumonia*e is a global public health concern since this antibiotic is the last defense line against carbapenem-resistant isolates [38]. This extreme resistance was confirmed in a recent study in Egypt (82%) [32]. Surprisingly, a lower resistance to amoxicillin/clavulinic acid (25%) was observed in this study. Similarly, in Egypt, a lower resistance (19%) to this antibiotic was reported [35], however, higher resistance to this antibiotic was observed in recent studies in Egypt; [32] (100%) and [34] (94.6%).

In this investigation, cephalosporin resistance of *K. pneumoniae* isolates to ceftriaxone (50%) and cefotaxime (90%) was detected. However, a lower resistance to cefotaxime (5%) was reported in Egypt [35]. Moreover, in the present work, a moderate resistance to fluoroquinolones (45%) was noticed. It seems that nations that have prohibited or never used fluoroquinolones in poultry have substantially lower levels of resistance than countries that continue to use the antibiotics in poultry [39], where, fluoroquinolones still used in poultry sectors in Egypt [40].

The resistance of human isolates to imipenem, in this study, was 69.2%. Nearly similar imipenem resistance (64.5%) has recently been reported in Iran [41]. However, a lower imipenem resistance was reported earlier in Egypt by [42] (50%) [43], (31.3%), and [44] (3.5%). Furthermore, the resistance of human isolates to cefotaxime and ceftriaxone was 69.2%, each. In this regard, all of the 112 consecutive *K. pneumoniae* nosocomial isolates from Mansoura University Hospitals were resistant to the third generation cephalosporins [19]. Also, a high resistance against cephalosporins, with a resistance rate of 69.8% for ceftriaxone was noticed [44]. In its list of antimicrobial-resistant bacteria, the WHO has designated carbapenem-resistant and third-generation cephalosporin-resistant *Enterobacteriaceae* (including *K. pneumoniae*) to be one of the highest priority pathogens that necessitate the need of novel therapies [45].

The re-use of colistin (last-resort drug) to treat infections caused by resistant Gram-negative bacteria, has led to the emergence of a serious resistance against colistin [46]. In this study, phenotypic colistin resistance in human samples was very high (92.3%), however, in another study, all the tested *K. pneumoniae* isolates were sensitive to colistin [42]. Also, lower resistances for colistin had been recently reported (18.9%) [34] and (2.3%) [44]. Furthermore, the prevalence of colistin resistance among *K. pneumoniae* was 4%, 8%, 27.5%, and 31.4% in India, Kuwait, Turkey, and United Arab Emirates, respectively [47].

These variations in the resistance rates to different antimicrobial agents may be due to different criteria of patient selection, the case mix, severity of illness, type of ICU, stay durations, rate of device utilization, and criteria of discharge [48]. Generally, the incidence of resistance of bacteria against antibiotics can occur due to various factors, including the excessive long term use of antibiotics, with high doses causing a global crisis of antibiotic resistance. Besides, long-term overexposure to trace levels of antibiotics through contaminated foods can cause normal microflora in the digestive system to adapt and develop antibiotic resistance [49].

Overall, out of 33 *K. pneumoniae* tested strains, in the current work, MDR (33.3%), XDR (57.6%) and PDR (9.1%) were detected. Recently, the report of PDR and high percentage of XDR *K. pneumoniae* isolates is being increased. In Bangladesh, resistance pattern of *K. pneumoniae* yielded (39.1%) MDR, (21.7%) XDR, and (3.75%) PDR [50]. Additionally, in Iran, MDR (84.6%), XDR (13.5%) and PDR (1.9%) were detected [41]. However, in a recent study in Egypt, out of 23 tested isolates, MDR (82.6%), XDR (13.04%) were detected but no PDR isolates were identified [18]. Moreover, earlier investigations have reported that *K. pneumoniae* isolates from chicken were MDR [35, 51, 52].

The resistance gene profiling of the 33 strains revealed that, out of 20 broiler chicken isolates, the *cml*A (65%), *dfr*A1 (65%), *mcr*1 (60%), *tet*A (50%) were the most frequently encountered genes. A study in India showed that 51 (86.44%) *K. pneumoniae* isolates of different sources, including chickens, were found to harbor *sul*1 gene [53]. Moreover, *K. pneumoniae* isolated from healthy broiler chicken in India were resistant to tetracycline and harbored *tet*A gene in another study [54]. However, in a recent study from Europ, few antibiotic resistance genes were observed; *sul* and *dfr* (9.2%; 12/131, both) and *tet* (8.4%; 11/131) across 131 *K. pneumoniae* isolates from chicken meat and salad samples [55].

In South Africa, *fos*A gene (95%), *sul*1 (78%) and *dfr*A1 (33%), had been reported in *K. pneumoniae* clinical isolates, while *mcr*1 gene had not been detected [56]. Also, in Iran, *sul*1 (71%), *dfr* (29%) and *fos*A (40%) were detected at higher rates [57]. Also, *K. pneumoniae* colistin resistant strains from humans were carrying the *mcr-*1 gene [58]. However, contrary to findings in the present study, a lower prevalence of colistin-resistance had been observed earlier [46]. The authors suggest that it has not extended to the community yet and the *mcr-*1 gene was not detected in any phenotypically colistin-resistant isolates of their study [46]. The plasmid-mediated mobile colistin resistance (*mcr*) gene family (*mcr-*1 to *mcr-*10), was identified worldwide and poses a great threat to public health due to possible spreading of such resistnant strains through food chain, which facilitates rapid dissemination of colistin resistance [59]. Moreover, the emergence of carbapenemases together with the *mcr-*1 colistin resistance gene constitutes a global risk to public health [60].

Overall, it was not surprising that these genes were identified significantly in isolates from Egyptian patients since it was established in Egypt the frequent usage of aminoglycosides for the management of many respiratory infections such as chronic endobronchial infections [61]. Additionally, the profuse misuse of tetracycline in Egyptian poultry farms allows the spreading of tetracycline resistance through the poultry litter or droppings containing tetracycline [62].

The virulence gene profiling of *K. pneumoniae* isolates, in the present study, targeted the *rmp*A, *mag*A, and *fim*H genes. Franklin-Alming and co-authors [63] failed to detect *rmpA*-genes in healthy poultry samples. However, in China, lower percentages of *rmp*A (11.15) and *mag*A gene (8.9) were detected, and fimbrial adhesin gene *fim*H-1 was the most prevalent virulence gene (95.6%) [33]. Moreover, in an earlier study from Egypt, *K. pneumoniae* isolates were positive for *rmp*A gene (46.67%) and *mag*A gene (53.33%) [64]. The high occurrence of such virulence genes from apparently healthy broiler chicken strains, in the current study, shed the light on possible food safety concerns.

In human isolates, the *rmp*A gene detection rate was closely similar to that reported in *K. pneumoniae* isolates obtained from neonates with septicemia in Egypt (47.9%) [65], however it was lower than that reported in Tehran, Iran (83%) [66]. Additionally, the *mag*A gene percentage reported in the present study, was much higher than the two later mentioned studies. Moreover, the *fim*H gene was not detected among Egyptian selected *Klebsiella* clinical isolates in another study [67]. In Iran, 86.5% of *K. pneumoniae* isolates carried *fim*H gene [41]. Recent studies suggesting that the development of drug resistance of *K. pneumoniae* has been reported to boost virulence [5], and increased virulence is believed to naturally evolve in response to, or potentially be shared among bacteria, leading to emergence of resistance [68].

Determination of colistin resistance using usual disk diffusion method utilizing typical agar concentrations without any modifications or using MIC test, is a limitation in the present study. Data in the current work represent a preliminary step for a larger surveillance programs in the study area. Further studies with larger number of samples and advanced genomic techniques are needed to confirm the zoonotic potential posed of such isolates from apparently healthy broiler chicken.

## Conclusion

The phenotypic and genotypic profile of *K. pneumoniae* isolates from apparently healthy broiler chickens suggest a possible threat to public health due to movement of resistant strains through food chain. Moreover, occurrence of MDR *K. pneumoniae* human isolates, urgently suggest following strict measures for the uncontrolled usage of antibiotics.

## Data Availability

All data generated or analyzed during this study is included in this published article.
